# Genome sequence and description of the anaerobic lignin-degrading bacterium *Tolumonas lignolytica* sp. nov.

**DOI:** 10.1186/s40793-015-0100-3

**Published:** 2015-11-19

**Authors:** Andrew F. Billings, Julian L. Fortney, Terry C. Hazen, Blake Simmons, Karen W. Davenport, Lynne Goodwin, Natalia Ivanova, Nikos C. Kyrpides, Konstantinos Mavromatis, Tanja Woyke, Kristen M. DeAngelis

**Affiliations:** Microbiology Department, University of Massachusetts, Amherst, MA USA; Microbial Communities Group, Deconstruction Division, Joint BioEnergy Institute, Emeryville, CA USA; Department of Civil & Environmental Engineering, The University of Tennessee, Knoxville, TN USA; Department of Microbiology, The University of Tennessee, Knoxville, TN USA; Department of Earth & Planetary Sciences, The University of Tennessee, Knoxville, TN USA; Sandia National Lab, Livermore, CA USA; Los Alamos National Laboratory, Los Alamos, NM USA; Department of Energy Joint Genome Institute, Walnut Creek, CA USA

**Keywords:** Anaerobic lignin degradation, Tropical forest soil isolate, *Tolumonas*

## Abstract

*Tolumonas lignolytica* BRL6-1^T^ sp. nov. is the type strain of *T. lignolytica* sp. nov., a proposed novel species of the *Tolumonas* genus. This strain was isolated from tropical rainforest soils based on its ability to utilize lignin as a sole carbon source. Cells of *Tolumonas lignolytica* BRL6-1^T^ are mesophilic, non-spore forming, Gram-negative rods that are oxidase and catalase negative. The genome for this isolate was sequenced and returned in seven unique contigs totaling 3.6Mbp, enabling the characterization of several putative pathways for lignin breakdown. Particularly, we found an extracellular peroxidase involved in lignin depolymerization, as well as several enzymes involved in β-aryl ether bond cleavage, which is the most abundant linkage between lignin monomers. We also found genes for enzymes involved in ferulic acid metabolism, which is a common product of lignin breakdown. By characterizing pathways and enzymes employed in the bacterial breakdown of lignin in anaerobic environments, this work should assist in the efficient engineering of biofuel production from lignocellulosic material.

**Electronic supplementary material:**

The online version of this article (doi:10.1186/s40793-015-0100-3) contains supplementary material, which is available to authorized users.

## Introduction

The exponential increase in anthropogenic greenhouse gas emissions following the industrial revolution has drastically affected the climate of Earth, inspiring the need to produce clean, renewable energy with the goal of mitigating the consequences of burning fossil fuels. Second generation biofuels are a promising source of sustainable energy because they are derived from lignocellulose, the most abundant natural polymer on Earth. However, this material is highly recalcitrant due to the occlusion of cellulose by lignin, and the microbial pathways for lignin degradation are not yet well understood.

Lignin is a complex aromatic heteropolymer present in the cell wall of all plants, and comprises 10–30 % of cell wall material [1]. Lignin forms intricate associations with cellulose, the most abundant component within the cell wall, and serves as defense for plants, blocking access of cellulase enzymes to resist microbial breakdown. Consequently, the production of biofuels from plant biomass is physically and chemically hindered by lignin and its links to cellulose [1]. Aerobic lignin degradation has been extensively studied in fungi, suggesting that lignolytic extracellular peroxidase and laccase enzymes play a significant role in the mineralization of lignin in soil [2, 3]. Recent studies focusing on bacterial breakdown and modification of lignin have found that members of the phylogenetic groups *Alphaproteobacteria*, *Gammaproteobacteria*, *Firmicutes* and *Actinomycetes* are major players in lignin degradation, in both soil and insect guts [4]. Among bacterial lignin or phenol degraders, *Sphingomonas paucimobilis* SYK-6 produces a β-aryl etherase [5], and *Rhodococcus* sp. RHA1 contains a β-ketoadipate pathway [6]; *Kocuria* and *Staphylococcus* also likely degrade aromatic compounds derived from lignocellulose [7]. Although many lignolytic bacteria grow in environments where oxygen is depleted [8], it has been suggested that they employ oxygen-requiring peroxidases, similar to the ones utilized by fungi [9].

To address the need for more efficient removal of the lignin portion of lignocellulose to streamline biofuel production, we isolated anaerobic bacteria from tropical rainforest soil in Puerto Rico. Humid tropical forest soils like those from the Long Term Ecological Research Station at the Luquillo Experimental Forest in Puerto Rico have been shown to have among the fastest rates of plant litter decomposition globally [10], despite their low and fluctuating redox potential [11]. Frequent episodes of anoxia at the Luquillo Forest inhibit fungal growth [12], suggesting that bacteria are responsible for the observed litter decomposition, and consequently providing an optimal environment for isolating bacteria involved in the anaerobic decomposition of plant litter, including cellulose and lignin compounds. Bacteria are more amenable to genetic modification than fungi and thus are more easily incorporated into biofuel processing technology, for instance through metabolic engineering. Additionally, bacteria capable of metabolizing lignin anaerobically are favorable to industrial biofuel production, considering that current technology relies on anaerobic digestors to process plant waste into biofuels [13]. With this in mind, we isolated and characterized a bacterium capable of anaerobic lignin degradation, *Tolumonas lignolytica*BRL6-1^T^ sp. nov., and provide a summary of its genome sequence and annotation.

## Organism information

### Classification and features

*Tolumonas lignolytica*BRL6-1^T^ was isolated from soil collected at the Bisley watershed site of the El Yunque National Forest in Puerto Rico, part of the Luquillo Experimental Forest in Luquillo, Puerto Rico, USA (Table 1; Additional file 1). Soils were diluted in water and used to inoculate anaerobic roll tubes containing a modified CCMA media consisting of 2.8 g L^−1^ NaCl, 0.1 g L^−1^ KCl, 27 mM MgCl_2_, 1 mM CaCl_2_, 1.25 mM NH_4_Cl, 9.76 g L^−1^ MES, 1.1 ml L^−1^ K_2_HPO_4_, 12.5 ml L^−1^ trace minerals [14, 15], and 1 ml L^−1^ Thauer’s vitamins [16], with alkali lignin added as the sole source of carbon. Tubes were incubated at ambient temperature for 3 months before colonies were picked and characterized.

**Table 1:** Classification and general features of *Tolumonas lignolytica* BRL6-1

MIGS ID	Property	Term	Evidence code^a^
	Classification	Domain *Bacteria*	TAS [45, 46]
		Phylum *Proteobacteria*	TAS [47]
		Class *Gammaproteobacteria*	TAS [48]
		Order *Aeromonadales*	TAS [49]
		Family *Aeromonadaceae*	TAS [50]
		Genus *Tolumonas*	TAS [51]
		Species *Tolumonas lignolytica*	
		Strain BRL6-1^T^	
	Gram stain	negative	IDA
	Cell shape	rod	IDA
	Motility	motile	IDA
	Sporulation	non-sporulating	IDA
	Temperature range	15–37 °C	IDA
	Optimum temperature	30 °C	IDA
	pH range; Optimum	4.5–8.5; 7	
	Carbon source	glucose, lactose, others (Table 6)	IDA
MIGS-6	Habitat	Tropical forest soil	TAS [52]
MIGS-6.3	Salinity	1 % NaCl	IDA
MIGS-22	Oxygen requirement	Facultative aerobe	IDA
MIGS-15	Biotic relationship	Free-living	IDA
MIGS-14	Pathogenicity	Non-pathogenic	NAS
MIGS-4	Geographic location	Soil collected from a subtropical lower montane wet forest in the Luquillo Experimental Forest, part of the NSF- sponsored Long-Term Ecological Research program in Puerto Rico	IDA
MIGS-5	Sample collection	July 2009	IDA
MIGS-4.1	Latitude	18.268 N	IDA
MIGS-4.2	Longitude	65.760 W	IDA
MIGS-4.4	Altitude	375 m	IDA

^a^Evidence codes - *IDA* Inferred from Direct Assay, *TAS*, Traceable Author Statement (i.e., a direct report exists in the literature), *NAS* Non-traceable Author Statement (i.e., not directly observed for the living, isolated sample, but based on a generally accepted property for the species, or anecdotal evidence). These evidence codes are from the Gene Ontology project [53]

**Fig. 1:**
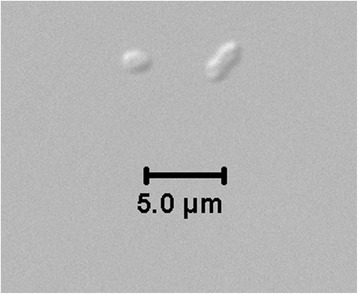
Photomicrograph of *Tolumonas lignolytica* BRL6-1^T^. The sample was prepared by growing a culture overnight in 10 % Tryptic Soy Broth (TSB), then adhering to microscope slide coverslips that were treated with poly-lysine to facilitate attachment of cells. The image was taken with a Nikon E500 Fluorescence Microscope

*Tolumonas**lignolytica*BRL6-1^T^ cells are Gram-negative, facultative rods capable of growth from 15 to 37 °C and pH 4.5–8.5 (Fig. 1). Colonies on 10 % Tryptic Soy Agar (TSA) plates appear as white, flat circles with opaque centers and translucent halos.

**Fig. 2:**
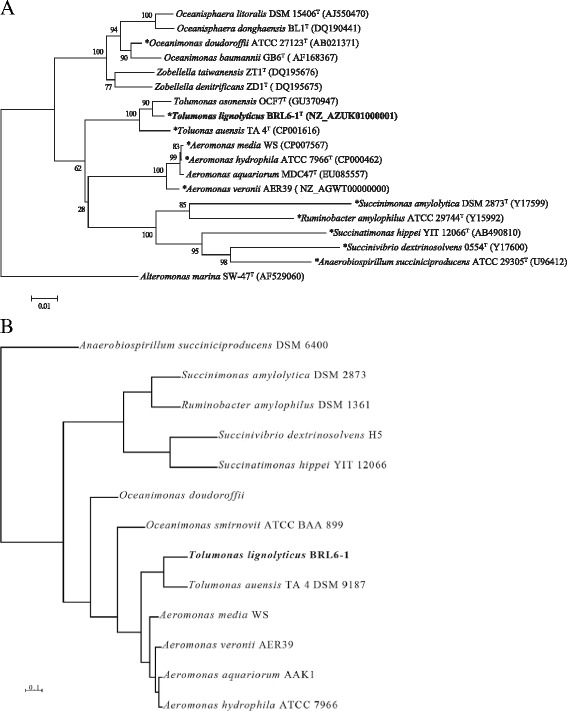
Phylogenetic tree highlighting the position of Tolumonas lignolytica BRL6-1^T^ among the Aeromonadales. **a** The phylogenetic tree based on 16S ribosomal RNA gene sequence was inferred using the Neighbor-Joining method [54] within MEGA6 [55]. Bootstrap values of 1000 replicate trees are shown at the branches [56]. The tree is drawn to scale, with branch lengths in the same units as those of the evolutionary distances used to infer the phylogenetic tree. The evolutionary distances were computed using the Jukes-Cantor method [57] and are in units of the number of base substitutions per site. All positions containing gaps and missing data were eliminated, creating a total of 1234 positions in the final dataset. GenBank accession numbers are shown in *parentheses* after strain numbers. Type strains are indicated with a *superscript* T. Organisms with genomes available are indicated by an *asterisk* before the name. **b** Whole genomes were hierarchically clustered based on COG profiles using tools in IMG [58]. T. lignolytica BRL6-1^T^ is indicated in *bold* in both phylogenetic trees

Sanger sequencing was performed on the small subunit ribosomal RNA (16S rRNA) gene using universal primers 27F and 1492R [17]. BLAST analysis shows 97 % identity to the full length 16S rRNA gene of *Tolumonas auensis* type strain TA 4, indicating BRL6-1^T^ as a potentially novel species of the *Tolumonas* genus, within the *Aeromonadaceae* family of the *Gammaproteobacteria* (Fig. 2a). Since the 16S rRNA gene sequence is not sufficient to clearly define the evolutionary history of this cluster of the *Gammaproteobacteria*, a hierarchical clustering of whole genomes based on COGS was constructed [18] (Fig. 2b). This clustering supports the placement of *T. lignolytica*BRL6-1^T^ as a novel species within the *Tolumonas* genus.

## Genome sequencing information

### Genome project history

*Tolumonas lignolytica*BRL6-1^T^ was selected for sequencing based on its ability to utilize lignin as a sole carbon source. Genome sequencing was performed by the JGI and completed on February 22, 2013 and the genome was presented for public access on IMG M/ER on August 28, 2013. Table 2 presents the project information and its association with MIGS version 2.0 compliance [19].

**Table 2:** Sequencing project information

MIGS ID	Property	Term
MIGS-31	Finishing quality	High-quality draft
MIGS-28	Libraries used	Three libraries: Illumina std shotgun library, Illumina long insert mate pair library, Pacbio SMRTbell™ library
MIGS-29	Sequencing platforms	Illumina HiSeq 2000, PacBio RS
MIGS-31.2	Fold coverage	2680X, 1157X, 33X
MIGS-30	Assemblers	AllpathsLG
MIGS-32	Gene calling method	Prodigal, GenePRIMP
	Locus Tag	H027
	GenBank ID	AZUK00000000.1
	GenBank Date of Release	May 23, 2014
	GOLD ID	Gi0037033
	BIOPROJECT	PRJNA186459
MIGS-13	Source Material Identifier	In submission
	Project relevance	Biofuels, Environmental

### Growth conditions and genomic DNA preparation

For genomic DNA extraction, strain BRL6-1^T^ was grown overnight in 10 % TSB at 30 °C with shaking at 225 rpm. Genomic DNA for sequencing was obtained following a modified cetyl-trimethylammonium bromide (CTAB) extraction protocol established by the DOE Joint Genome Institute. Modifications were as follows: 1) Overnight cultures were resuspended to an OD@600 nm of 0.5, instead of 1.0; 2) Lysozyme incubation was carried out at 37 °C for 30 min; 3) Proteinase K incubation was carried out for 3 h; 4) the concentration of Proteinase K was doubled. The extracted DNA was quantified using the Invitrogen™ Quant-iT™ PicoGreen® dsDNA Assay Kit and measured using the PicoGreen Fluorescence protocol on the SpectraMax M2 Microplate Reader by Molecular Devices. Genomic DNA samples were verified as strain BRL6-1^T^ via 16S rRNA gene sequencing before being shipped to JGI for genome sequencing.

### Genome sequencing and assembly

The draft genome of strain BRL6-1^T^ was generated at the DOE Joint Genome Institute using both Illumina and Pacific Biosciences (PacBio) technologies. An Illumina standard shotgun library and long insert mate pair library was constructed and sequenced using the Illumina HiSeq 2000 platform [20]. 64,682,509 reads totaling 9702.4 Mb were generated from the standard shotgun and 45,878,643 reads totaling 4175.0 Mb were generated from the long insert mate pair library. A Pacbio SMRTbell™ library was constructed and sequenced on the PacBio RS platform. 41,131 raw PacBio reads yielded 41,162 adapter-trimmed and quality filtered subreads totaling 118.4 Mb. All raw Illumina sequence data was passed through DUK, a filtering program developed at JGI, which removes known Illumina sequencing and library preparation artifacts [21]. Filtered Illumina and PacBio reads were assembled using AllpathsLG as previously described [22]. The total size of the genome is 3.6 Mb. The final draft assembly contained 9 contigs in 9 scaffolds, and was based on 9669.4 Mb of Illumina Std PE, 4174.1 Mb of Illumina CLIP PE and 118.4 Mb of PacBio post filtered data, which provides an average 3837X Illumina coverage and 32.8X PacBio coverage of the genome, respectively.

### Genome annotation

Genes were identified using Prodigal [23], followed by a round of manual curation using GenePRIMP [24]. The predicted CDSs were translated and used to search the National Center for Biotechnology Information nonredundant database, UniProt, TIGRFam, Pfam, KEGG, COG, and InterPro databases. The tRNAScanSE tool [25] was used to find tRNA genes, whereas ribosomal RNA genes were found by searches against models of the ribosomal RNA genes built from SILVA [26]. Other non-coding RNAs such as the RNA components of the protein secretion complex and the RNase P were identified by searching the genome for the corresponding Rfam profiles using INFERNAL [27]. Additional gene prediction analysis and manual functional annotation was performed within the Integrated Microbial Genomes (IMG) platform [28] developed by the Joint Genome Institute, Walnut Creek, CA, USA [29].

## Genome properties

**Fig. 3:**
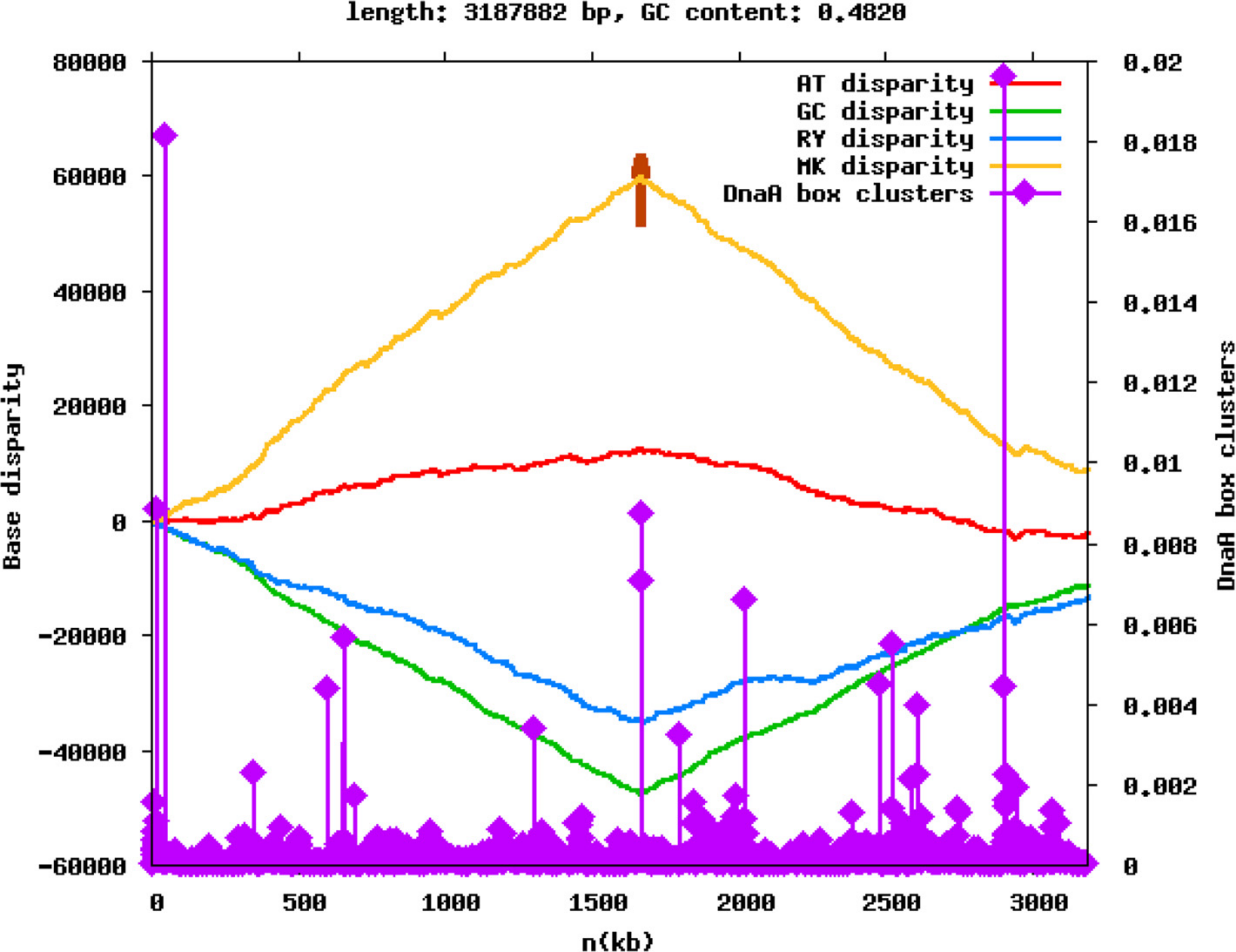
Z-curve graph of *T. lignolytica* BRL6-1^T^ contig 1. The Z-curve graph was made using Ori-Finder software. The sequence is rotated such that the beginning and ending are the maximum of the GC disparity curve. The *short vertical red line* indicates the location of the indicator gene dnaA, while *purple peaks* with *diamonds* indicate DnaA box clusters. The shift in base pair disparity coincides with the location of the dnaA gene and a large cluster of DnaA boxes, which together strongly suggest the location of the origin of replication. MK disparity represents the ratio of amino (A,C) to keto (G,T) bases, while RY disparity represents the ratio of purine (A,G) to pyrimidine (C,T) bases. Finally, the AT and GC disparities represent the ratio of weak to strong hydrogen bond forming bases, respectively

One chromosomal origin of replication, located at position 1,760,186–1,760,733 bp of contig 1 was identified, suggesting that the genome contains only one chromosome of at least 3.187Mbp (Fig. 3). The location was determined using the Z-curve method [30–32], which utilizes base pair disparities to create a unique three-dimensional graph of the genome using Ori-Finder software [33]. Although nine contigs are presented in the GenBank record (for the genome AZUK00000000.1), contigs 5 and 9 were direct repeats of sequences contained in the other contigs, so we hypothesized that they are repeated scaffolds and excluded them from our analyses (Table 3). Due to remarkably high repeat content at the ends of contigs, we were unable to close the gaps between them using regular sequencing methods. The contigs may be part of the chromosome, but a plasmid extraction indicated the presence of at least one plasmid. A search through the PATRIC database of plasmid sequences shows that contigs 2–8, excluding 5, all have homology to known plasmid sequences, using maximum E-value of 1e^−5^ [34]. Furthermore, contigs 3,4,7, and 8 have annotated genes commonly found in plasmids, such as toxin-antitoxin sequences, prevent-host-death family sequences, and plasmid maintenance and stabilization protein genes, making them likely candidates.

**Table 3:** Summary of genome: one chromosome and six other contigs

Label	Size (kb)	Topology	INSDC identifier
Chromosome 1	3187	linear	AZUK01000001
Contig 2	316	linear	AZUK01000002
Contig 3	44	linear	AZUK01000003
Contig 4	24	linear	AZUK01000004
Contig 6	12	linear	AZUK01000006
Contig 7	11	linear	AZUK01000007
Contig 8	10	linear	AZUK01000008

Of the 3427 predicted genes, 3323 were identified as protein-encoding genes, while 131 RNA genes were found. Of the total protein coding genes identified, 75.02 % were assigned to a putative function. The properties and the statistics of the genome are summarized in Tables 3, 4 and 5.

**Table 4:** Nucleotide content and gene count levels of the genome

Attribute	Value	% of total
Genome size (bp)	3,607,052	100.00
DNA coding (bp)	3,268,621	89.99
DNA G+C (bp)	1,715,377	47.56
DNA scaffolds	7	100.00
Total genes	3427	100.00
Protein coding genes	3296	96.18
RNA genes	131	3.82
Pseudo genes	24	0.70
Genes in internal clusters	2431	70.94
Genes with function prediction	2571	75.02
Genes assigned to COGs	2797	81.62
Genes with Pfam domains	2916	85.09
Genes with signal peptides	273	7.97
Genes with transmembrane helices	755	22.03
CRISPR repeats	1	–

The total is based on either the size of the genome in base pairs or the total number of protein coding genes in the annotated genome

**Table 5:** Number of genes associated with COG functional categories

Code	Value	% age	Description
J	168	5.10	Translation, ribosomal structure and biogenesis
A	1	0.03	RNA processing and modification
K	217	6.58	Transcription
L	129	3.91	Replication, recombination and repair
B	0	0.00s	Chromatin structure and dynamics
D	35	1.06	Cell cycle control, cell division, chromosome partitioning
V	37	1.12	Defense mechanisms
T	171	5.19	Signal transduction mechanisms
M	159	4.82	Cell wall/membrane biogenesis
N	112	3.40	Cell motility
U	88	2.67	Intracellular trafficking and secretion
O	106	3.22	Posttranslational modification, protein turnover, chaperones
C	176	5.34	Energy production and conversion
G	247	7.49	Carbohydrate transport and metabolism
E	241	7.31	Amino acid transport and metabolism
F	73	2.21	Nucleotide transport and metabolism
H	144	4.37	Coenzyme transport and metabolism
I	50	1.52	Lipid transport and metabolism
P	126	3.82	Inorganic ion transport and metabolism
Q	31	0.94	Secondary metabolites biosynthesis, transport and catabolism
R	259	7.86	General function prediction only
S	245	7.43	Function unknown
–	930	28.21	Not in COGs

The total is based on the total number of protein coding genes in the annotated genome

## Insights from the genome sequence

### Genome to genome comparisons

Once the genome of strain BRL6-1^T^ was sequenced, we were able to compare it to the genome of *T. auensis*, its closest relative. The two genomes have an average nucleotide identity (ANI) of 84 %, far below the 95 % threshold for species delineation [35]. A tool developed by DSMZ called the genome to genome distance calculator (GGDC) compares genome sequences to databases of DNA-DNA hybridization (DDH) data [36]. It estimates the DDH between these two genomes to be 23.10 % +/− 2.37, again far below the species threshold (70 %). The GGDC also uses logistic regression to estimate the probability that DDH > 70 %, i.e. the two genomes belong to the same species. The GGDC calculated a <0.01 % chance that DDH > 70 % between the genomes of strain BRL6-1^T^ and *T. auensis**.* These data support the assertion that strain BRL6-1^T^ is a novel species.

### Oxidative enzymes

**Fig. 4:**
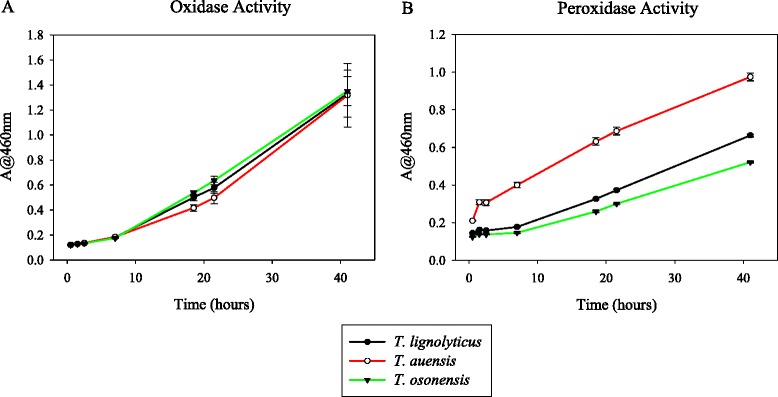
Oxidase and peroxidase enzyme activity for three *Tolumonas* organisms. Oxidative enzyme assays were performed by incubating isolates in nutrient broth with 25 mM L-DOPA. Peroxidase enzyme assays were performed in the same manner, with the addition of 3 % hydrogen peroxide. A negative control without inoculum was also measured and subtracted from experimental values

To facilitate physiological comparisons among the species within the *Tolumonas* genus, *T. auensis* and *T. osonensis* were acquired from the DSMZ culture collection in Germany. Oxidase tests confirmed that all three *Tolumonas* species, including *Tolumonas**lignolytica*BRL6-1^T^, are negative for oxidation of cytochrome c, as colonies did not change color when applied to BD BBL™ DrySlide™ test strips. However, oxidative enzyme assays show that all three organisms are capable of oxidizing 3,4-dihydroxy-L-phenylalanine (L-DOPA), a compound that is utilized as a lignin analog (Fig. 4a).

A catalase test was performed on each *Tolumonas* organism by dropping 3 % hydrogen peroxide on cultures that had incubated at 30 °C for 20 h. *T. lignolytica* and *T. auensis* had very weak positive reactions, while *T. osonensis* exhibited a much stronger phenotype. However, all three organisms showed relatively strong peroxidase enzyme activity (Fig. 4B). A search through the genomes of *T. auensis* and *T. lignolytica* shows that both organisms possess just one copy of the same enzyme with putative catalase activity (EC:1.11.1.21), while each genome contains several peroxidase genes. *T. osonensis* does not have its genome sequenced and thus we could not search for catalase or peroxidase genes.

### Carbon utilization

Based on the genome sequence of *T. lignolytica*BRL6-1^T^, we expected that this organism would be able to easily utilize fructose, glucose, mannitol, sorbitol, sucrose, and trehalose, as there are phosphotransferase system (PTS) genes annotated specifically for these carbon sources. Biolog phenotypic arrays were used to test the carbon sources utilized by strain BRL6-1^T^ under anaerobic conditions, using a modified version of DSMZ medium 500 (referred to as mGV medium), in which FeSO_4_ · 7H_2_O, Na_2_S · 9H_2_O, yeast extract, and the selenite/tungstate solution were omitted. Plates were performed in duplicate, and reactions were only considered positive if the difference between average and standard error was greater than 20 units. Our results indicated that out of the 96 carbon sources tested, strain BRL6-1^T^ was able to utilize 26 under anaerobic conditions (Table 6), included the carbon sources predicted based on the genome sequence. Summarized in Table 7 are carbon sources that are differentially utilized among the three *Tolumonas* organisms under anaerobic conditions [37, 38].

**Table 6:** Carbon sources utilized by *Tolumonas lignolytica* BRL6-1^T^ anaerobically

Carbon source	Omnilog units
D-Trehalose	168
L-Arabinose	166
D-Fructose	164
D-Sorbitol	161
D-Gluconic Acid	161
a-D-Glucose	161
Maltotriose	156
N-Acetyl-D-Glucosamine	145
Sucrose	131
D-Galactose	128
Tween 20	128
Maltose	128
D-Mannose	108
Tween 40	106
D-Melibiose	102
D-Mannitol	95
D-Ribose	91
Tween 80	62
L-Lyxose	36

**Table 7:** Carbon sources differentially utilized by *Tolumonas* species anaerobically

Carbon source	*T. auensis*	*T. osonensis*	*T. lignolytica*
D-Arabinose	+	−	+
D-Lactose	−	+	+
D-Xylose	−	−	+
Glycerol	−	−	+
Fumarate	−	+	−
Pyruvate	−	+	−

We predicted that lactose fermentation would be possible in strain BRL6-1^T^, as the genome contains four copies of PTS system genes specific to lactose, more than for any other carbon source. *T. lignolytica*BRL6-1^T^ grows well on aerobic plates of eosin methylene blue agar (EMB) medium, and produces metallic green colonies after 2 days incubation, suggesting aggressive lactose fermentation, a common characteristic of members of the *Enterobacteriaceae* family. Considering that enteric bacteria are common inhabitants of soils [39], it is plausible that this phenotype originated from horizontal gene transfer. The type strains of *T. auensis* and *T. osonensis* were able to grow slowly on aerobic EMB plates, but did not produce metallic green colonies. Therefore, EMB plate growth morphology can be used to easily distinguish *T. lignolytica*BRL6-1^T^ from the other *Tolumonas* species.

### Lignocellulose degradation

The genome of *Tolumonas lignolytica*BRL6-1^T^ contains four putative peroxidase genes, which may be important in depolymerizing lignin in the environment [40]. The genome also contains homologues to *ligD* and *ligF*, genes characterized in *Sphingomonas paucimobilis* SYK-6 [41] that encode enzymes responsible for the cleavage of β-aryl ether bonds. This type of bond comprises approximately 50 % of the linkages among lignin monomers [9], thus its cleavage is crucial to lignocellulose breakdown. Furthermore, strain BRL6-1^T^ possesses homologues to genes involved in the pathway for transforming ferulic acid, a common lignin breakdown product, into vanillate, then into protocatechuate, and finally to β-ketoadipate (summarized in Table 8) [42, 43].

**Table 8:** Enzymes involved in lignin degradation with homologues in BRL6-1^T^ genome

Enzyme	Gene name	Characterized in	# of copies in genome	E value	Gene annotation in BRL6-1^T^ genome
Cα-dehydrogenase involved in b-aryl ether cleavage	ligD	*Sphingomonas paucimobilis* SYK-6	2	6e-12; 4e-10	1)3-oxoacyl-[acyl-carrier-protein] reductase (EC 1.1.1.100) 2)hypothetical protein
Beta-etherase	ligF	*Sphingomonas paucimobilis* SYK-6	1	4e-11	Glutathione S-transferase
Feruloyl-CoA synthetase	fcs	*Pseudomonas putida* strain KT2440	1	1e-10	Acyl-CoA synthetases (AMP-forming)/AMP-acid ligases II
Feruloyl-CoA hydratase/lyase	ferB	*Sphingomonas paucimobilis*	1	7e-15	1,4-Dihydroxy-2-naphthoyl-CoA synthase (EC 4.1.3.36)
Feruloyl-CoA hydratase/lyase	ferB2	*Sphingomonas paucimobilis*	1	3e-32	1,4-Dihydroxy-2-naphthoyl-CoA synthase (EC 4.1.3.36)
Vanillin dehydrogenase	ligV	*Sphingomonas paucimobilis*	5	4e-66; 3e-65; 2e-62; 2e-35; 8e-08	1) succinate semialdehyde dehydrogenase (EC 1.2.1.16) 2) gamma-glutamyl-gamma-aminobutyraldehyde dehydrogenase 3) NAD-dependent aldehyde dehydrogenases 4) succinylglutamic semialdehyde dehydrogenase (EC 1.2.1.71) 5) acetaldehyde dehydrogenase (EC 1.2.1.10)/alcohol dehydrogenase AdhE (EC 1.1.1.1)
Vanillate O-demethylase oxidoreductase	vanB	*Pseudomonas syringae* pv. tomato strain DC3000	3	9e-18; 7e-14; 9e-12	1)Hemoglobin-like flavoprotein 2)Flavodoxin reductases (ferredoxin-NADPH reductases) family 1 3)Predicted ferric reductase
3-carboxymuconate cycloisomerase	pcaB	*Pseudomonas putida*	2	9e-13; 5e-09	1)Adenylosuccinate lyase (EC 4.3.2.2) 2)argininosuccinate lyase (EC 4.3.2.1)
4-carboxymuconolactone decarboxylase	pcaC	*Pediococcus acidilactici* D3	3	6e-30; 7e-27; 1e-06	Uncharacterized homolog of gamma-carboxymuconolactone decarboxylase subunit (all 3)
Β-ketoadipate enol-lactone hydrolase	pcaD	*Acinetobacter baumannii* strain SDF	2	5e-15; 5e-13	1)carboxylesterase BioH (pimeloyl-CoA synthesis) (EC 3.1.1.85) 2)Predicted hydrolases or acyltransferases (alpha/beta hydrolase superfamily)

Enzyme protein sequences were obtained from UniProt. E values are based on blastp results performed in img/er against the *T. lignolytica* BRL6-1^T^ genome

**Fig. 5:**
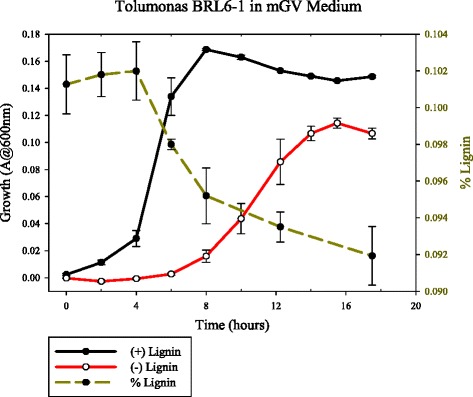
Growth curve of *Tolumonas lignolytica* BRL6-1^T^ in mGV medium. Solid lines depict the growth (*left axis*) of the organism with and without lignin amendment, with *error bars* showing the standard error of triplicate samples. The *dashed line* shows lignin concentration (*right axis*) throughout the growth curve. Lignin concentration was measured by removing 1 ml of culture from anaerobic septum bottles, diluting 1:10 in distilled water, filtering out cells, then measuring the absorbance at 310 nm. These values were compared to a standard curve of known concentrations of lignin in mGV medium measured at this wavelength

The genome also contains several cytochrome oxidase genes, which may be implicated in utilizing lignin as an electron acceptor for dissimilatory respiration, as was observed for ‘*gnolyticus*’ SCF1 [40], an organism that was obtained in the same isolation effort as BRL6-1^T^ [44]. Preliminary data supporting this hypothesis can be seen in Fig. 5, which depicts the growth of strain BRL6-1^T^ in a further simplified version of mGV medium (in which resazurin, sodium bicarbonate, and cysteine were also omitted from the recipe), with 0.2 % glucose supplied as a readily oxidized carbon source. The addition of lignin in the media increased both the growth rate and the maximum optical density achieved by strain BRL6-1^T^. Additionally, a decrease in lignin concentration correlates to exponential growth phase, suggesting that BRL6-1^T^ is using lignin as an additional carbon source, and/or as an electron acceptor, which may enhance the organism’s ability to utilize the more labile glucose as a carbon source*.*

## Conclusions

Based on biochemical characterization and genome analysis, we formally propose the creation of *Tolumonas lignolytica* sp. nov., of which BRL6-1^T^ is the type strain. Its 3.6 Mbp genome contains a suite of genes coding proteins involved in the breakdown of lignocellulosic material. These characteristics highlight its applicability to the industrial production of biofuels from plant biomass.

### Description of *Tolumonas lignolytica* sp. nov.

*Tolumonas*L. n. *lignum,* wood, lignin; Gr. Adj. *lytikos,* loosening, dissolving; NL fem adj. *lignolytia,* splitting lignin, referring to the ability to breakdown lignin).

The cells of type strain BRL6-1^T^ are Gram-negative rods that are oxidase and catalase negative. Colonies on 10 % TSA plates are flat white circles with opaque centers and characteristic translucent halos. Growth occurs at 15–37 °C with an optimum at 30 °C. The optimum pH is 7, and cells can tolerate up to 1 % NaCl. Grows well aerobically and anaerobically. The following carbon sources support anaerobic growth: d-trehalose, l-arabinose, d-fructose, d-sorbitol, d-gluconic acid, a-d-glucose, maltotriose, n-acetyl-d-glucosamine, sucrose, d-galactose, Tween 20, maltose, d-mannose, Tween 40, d-melibiose, d-mannitol, d-ribose, Tween 80, l-lyxose.

The G + C content of the genome is 47.56 %. The type strain BRL6-1^T^ (=DSM 100457 =ATCC [in submission]) was isolated from tropical rainforest soil using lignin as the sole carbon source.

## Additional file

Additional file 1: Table S1. Associated MIGS record. (DOC 74 kb)

## Abbreviations

RDP: Ribosomal Database Project (East Lansing, MI, USA)
CDS: Coding sequences
JGI: Joint Genome Institute (Walnut Creek, CA, USA)
