# Supplementary material for: Genome sequence and description of the anaerobic lignin-degrading bacterium Tolumonas lignolytica sp. nov
Source: Stand Genomic Sci. 2015 Nov 19;10:106. doi: 10.1186/s40793-015-0100-3 (PMC4653933; doi:10.1186/s40793-015-0100-3)
Supplement: Additional file 1: Table S1. — Associated MIGS record. (DOC 74 kb) [file 40793_2015_100_MOESM1_ESM.doc]

**Associated MIGS Record**

**Additional file 1: Table S1.** Associated MIGS record

| **MIGS-ID** | field name | description |
| --- | --- | --- |
| **MIGS-1** | Submit to INSDC/Trace archives | NZ_AZUK00000000.1 |
| **1.1** | PID |  |
| **1.2** | Trace Archive |  |
| **MIGS-2** | MIGS CHECK LIST TYPE | 2.0 |
| **MIGS-3** | Project Name | Four anaerobic lignin-degrading bacteria isolated from Puerto Rico tropical forest soils |
| **MIGS-4** | Geographic Location | Luquillo Experimental Forest |
| **4.1** | Latitude | 18.268 N |
| **4.2** | Longitude | 65.760 W |
| **4.3** | Depth | 10 cm |
| **4.4** | Altitude | 375 m |
| **MIGS-5** | Time of Sample collection | July 2009 |
| **MIGS-6** | Habitat (EnvO) | Tropical forest oil |
| **6.1** | temperature | 15°C-37°C |
| **6.2** | pH | 4.5-8.5 |
| **6.3** | salinity | 1% NaCl |
| **6.4** | chlorophyll |  |
| **6.5** | conductivity |  |
|
| **6.6** | light intensity |  |
| **6.7** | dissolved organic carbon (DOC) |  |
| **6.8** | current |  |
| **6.9** | atmospheric data |  |
| **6.10** | density |  |
| **6.11** | alkalinity |  |
| **6.12** | dissolved oxygen |  |
| **6.13** | particulate organic carbon (POC) |  |
| **6.14** | phosphate |  |
| **6.15** | nitrate |  |
| **6.16** | sulfates |  |
| **6.17** | sulfides |  |
| **6.18** | primary production |  |
| **MIGS-7** | Subspecific genetic lineage | [*Tolumonas*](http://dx.doi.org/10.1601/nm.3078) [BRL6](http://doi.org/10.1601/strainfinder?urlappend=%3Fid%3DBRL6)-1 |
| **MIGS-9** | Number of replicons | 1 |
| **MIGS-10** | Extrachromosomal elements | 0-6 |
| **MIGS-11** | Estimated Size | 3.6Mbp |
| **MIGS-12** | Reference for biomaterial or Genome report |  |
| **MIGS-13** | Source material identifiers |  |
| **MIGS-14** | Known Pathogenicity | Non-Pathogenic |
|
| **MIGS-15** | Biotic Relationship |  |
| **MIGS-16** | Specific Host |  |
| **MIGS-17** | Host specificity or range (taxid) |  |
| **MIGS-18** | Health status of Host |  |
| **MIGS-19** | Trophic Level |  |
| **MIGS-22** | Relationship to Oxygen | Facultative |
| **MIGS-23** | Isolation and Growth conditions |  |
| **MIGS-27** | Nucleic acid preparation | Modified CTAB protocol |
| **MIGS-28** | Library construction | Illumina std shotgun library; Illumina long insert mate pair library; Pacbio SMRTbell™ library |
| **28.1** | Library size | 150bp; 91bp; 2876bp |
| **28.2** | Number of reads | 64,682,509; 45,878,643; 41,162 |
| **28.3** | vector |  |
| **MIGS-29** | Sequencing method | Illumina HiSeq 2000, PacBio RS |
| **MIGS-30** | Assembly | Hybrid de novo |
| **30.1** | Assembly method | AllpathsLG |
| **30.2** | estimated error rate |  |
| **30.3** | method of calculation |  |
| **MIGS-31** | Finishing strategy | Not finished |
| **31.1** | Status | High-quality draft |
| **31.2** | coverage | 2680X , 1157X, 33X |
| **31.3** | contigs | 7 |
| **MIGS-32** | Relevant SOPs |  |
| **MIGS-33** | Relevant e-resources |  |
